# The Role of Zinc in Male Fertility

**DOI:** 10.3390/ijms21207796

**Published:** 2020-10-21

**Authors:** Deborah Allouche-Fitoussi, Haim Breitbart

**Affiliations:** The Mina and Everard Faculty of Life Sciences, Bar-Ilan University, Ramat-Gan 5290002, Israel; deboa743@gmail.com

**Keywords:** reproduction, zinc, spermatozoa, motility, capacitation, acrosome reaction

## Abstract

Several studies proposed the importance of zinc ion in male fertility. Here, we describe the properties, roles and cellular mechanisms of action of Zn^2+^ in spermatozoa, focusing on its involvement in sperm motility, capacitation and acrosomal exocytosis, three functions that are crucial for successful fertilization. The impact of zinc supplementation on assisted fertilization techniques is also described. The impact of zinc on sperm motility has been investigated in many vertebrate and invertebrate species. It has been reported that Zn^2+^ in human seminal plasma decreases sperm motility and that Zn^2+^ removal enhances motility. Reduction in the intracellular concentration of Zn^2+^ during epididymal transit allows the development of progressive motility and the subsequent hyper activated motility during sperm capacitation. Extracellular Zn^2+^ affects intracellular signaling pathways through its interaction with the Zn^2+^ sensing receptor (ZnR), also named GPR39. This receptor was found in the sperm tail and the acrosome, suggesting the possible involvement of Zn^2+^ in sperm motility and acrosomal exocytosis. Our studies showed that Zn^2+^ stimulates bovine sperm acrosomal exocytosis, as well as human sperm hyper-activated motility, were both mediated by GPR39. Zn^2+^ binds and activates GPR39, which activates the trans-membrane-adenylyl-cyclase (tmAC) to catalyze cAMP production. The NHE (Na^+^/H^+^-exchanger) is activated by cAMP, leading in increased pHi and activation of the sperm-specific Ca^2+^ channel CatSper, resulting in an increase in [Ca^2+^]_i_, which, together with HCO_3_^−^, activates the soluble adenylyl-cyclase (sAC). The increase in [cAMP]_i_ activates protein kinase A (PKA), followed by activation of the Src-epidermal growth factor receptor-Pphospholipase C (Src-EGFR-PLC) cascade, resulting in inositol-triphosphate (IP_3_) production, which mobilizes Ca^2+^ from the acrosome, causing a further increase in [Ca^2+^]_i_ and the development of hyper-activated motility. PKA also activates phospholipase D1 (PLD1), leading to F-actin formation during capacitation. Prior to the acrosomal exocytosis, PLC induces phosphadidylinositol-4,5-bisphosphate (PIP_2_) hydrolysis, leading to the release of the actin-severing protein gelsolin to the cytosol, which is activated by Ca^2+^, resulting in F-actin breakdown and the occurrence of acrosomal exocytosis.

## 1. Introduction

Zinc is an essential element with a wide range of biological functions. More than 200 Zn-metalloenzymes are regulated by Zn^2+^. It plays an essential role in the organization of DNA, RNA and proteins, as well as in the stability of cell membranes and cell division [1]. We know today that zinc is important for health. It is present in several organs like the kidney, skin, eyes, lung, brain, heart and pancreas at relatively high concentrations. It has been demonstrated that zinc ion assists immune function, helps cells to grow and proliferate in a healthy way and preserves prostate and sexual health. Zn^2+^ is required in diverse mechanisms, like the DNA replication, RNA transcription, differentiation, proliferation and activation of immune cells in many organs of the body [2,3,4]. In addition, zinc impacts on genes expressions have been reported. The structure of zinc finger domains found in transcription factors and regulating genes activity are also modulated by zinc [5]. Zinc ions also help to keep appropriated thyroid functions by creating “thyroid releasing hormones” in the brain that impact metabolism and development in the body. Low levels of zinc may affect the good production of these hormones, as well as testosterone levels [6].

## 2. Zinc and fertility

It has also been shown that zinc plays a significant role in the male reproductive system [7,8,9,10]. It was reported that fertile groups had higher levels of zinc in comparison to infertile groups. The level of Zn^2+^ in fertile men was 14.08± 2.01 and, in the infertile group, was 10.32 ± 2.98 (mg/100 mL) [11]. A Zn^2+^ lack can be linked with a diminution in testis volume and testicular weight and failure of spermatogenesis [12]. The presence of bacterial contamination in male and female reproductive tracts may negatively affect sperm function [13]. We know also that zinc has antibacterial activity: Zinc oxide nanoparticles kill both Gram-positive and Gram-negative bacteria and are also effective against spores, which are high temperature and high pressure-resistant [14,15]. The prostatic fluid of healthy men with high zinc affects mammalian spermatozoa after ejaculation [16]. The presence of zinc in semen was first described in 1921 [17]. Since then, many studies were performed to understand the role of zinc in male reproduction. The concentration of seminal zinc is associated with sperm count [18,19], and zinc-deficient nutrition causes a low quality of sperm and male infertility [11]. Rats treated with zinc show an increase in sperm count, sperm motility and testosterone levels and improved testicular structure and spermatogenesis abnormalities caused by obesity [20]. However, other studies claim that there is no significant association between zinc and sperm quality [21,22]. Zinc is found in spermatozoa and in the seminal fluid, where its concentration is higher than in all other body fluids. In semen, Zn^2+^ is secreted mainly from the prostate [23]. Zinc ion has been linked with key events in the acquisition of fertilization ability by spermatozoa, including motility, capacitation and acrosomal exocytosis. To enable fertilization, sperm must reside in the female reproductive tract for several hours, during which time, they undergo a series of biochemical and motility changes collectively called capacitation, allowing the spermatozoon to interact with the oocyte, undergo acrosomal exocytosis, and, finally, penetrate the egg. Defects in sperm quantity, quality or motility account for up to 50% of infertility cases and may affect approximately 7% of all men [24]. About 25% of infertility cases in humans are defined as “unexplained infertility”, and in many cases, successful fertilization in these men can be achieved by the technique of intracytoplasmic sperm injection (ICSI). On the other hand, in a non-negligible fraction of these unexplained cases, despite normal sperm quantity, morphology and motility, no egg penetration/fertilization occurs. Thus, it is possible that a significant proportion of unexplained infertility is, in fact, caused by spermatozoon failure to perform proper capacitation. It was shown that zinc deficiency is correlated with a decrease in male fertility [25,26], and the presence of zinc in the diets of humans [27] and domestic animals [28,29,30] is required for the achievement of an optimal fertility rate. The addition of Zn^2+^ to semen extenders before freezing reduces reactive oxygen species (ROS) [31]; however, excess Zn^2+^ can act as a pro-oxidant, leading to mitochondrial oxidative stress [32]. Structurally, the sperm mitochondrial sheath [33,34] and sperm chromatin [35,36] are stabilized by zinc bridges. The nuclear chromatin of mammalian sperm undergoes high condensation during the latter stages of spermatogenesis. This process is accomplished by a replacement of histones by the more basic amino acids arginine and cysteine-rich protamines. Zinc plays an essential role in nuclear chromatin decondensation after fertilization [37]. Relatively high concentrations of zinc inhibit chromatin decondensation in ram sperm, and the reverse situation was observed by chelating this Zn^2+^ [38]. Thus, the decrease in sperm Zn^2+^ during the epididymal transit is essential for the development of progressive motility, as well as chromatin decondensation, two necessary events for successful fertilization.

In this review, we will focus on the effect of Zn^2+^ on sperm motility, capacitation and acrosomal exocytosis, including the mechanisms of action and the impact of zinc supplementation on assisted fertilization techniques.

### 2.1. Regulation of Intracellular Zn^2+^ Concentrations

Two types of Zn^2+^ transporters are conserved in mammals, SLC39s/ZIPs and SLC30s/ZnTs, which transport Zn^2+^ in opposite directions through cellular and intracellular membranes (rev. by [39]). ZIPs increase the zinc concentration in the cytosol. For this, the ZIPs carry the zinc from extracellular and intracellular compartments to the cytosol. ZnTs reduce the concentration of zinc in the cytosol. For this, ZnTs carry the zinc from the cytosol to extracellular and intracellular compartments. After being transported to the cell, 50% of zinc is found in the cytoplasm, 30–40% in the nucleus and 10% in the plasma and organelle membranes (rev. by [40]). ZIP and ZnT have two major conformations, the inward-open and outward-open conformations (rev. by [39]). Based on sequence analyses, ZIP family members contain eight transmembrane domains as membrane transport proteins, with a cytoplasmic region between TM3 and TM4 [41,42]. The ZIP2 activity was significantly stimulated by HCO_3_—a treatment with high affinity to the first zinc and the next cadmium, suggesting a Zn^2+^/ HCO_3_-symporter mechanism [43]. ZnTs belong to a superfamily of cation diffusion facilitators observed in a wide range of taxa, including plants, bacteria, and fungi [38]. In mammals, at least 10 ZnT members have been identified [44,45]. They are predicted to have six trans-membrane domains (TMDs), except for ZnT5, which has additional TMDs, and their N- and C-termini face the cytoplasm, unlike those of ZIPs [44,45]. In ZnTs, zinc efflux occurs via a Zn^2+^/H^+^ antiporter [46]. This transporter mediates Zn^2+^ efflux to the outside of cells or to the luminal side of the intracellular compartment. At the same time, H^+^ ions are transported into the cytoplasm. Therefore, this transporter requires a proton motive force to mediate the Zn^2+^ efflux.

Investigations in *Caenorhabditis elegans* identified several genes that resulted in phenotypes of defective spermatogenesis (*spe*) and fertilization when mutated [47,48]. For example, the *spe*-8gene, which encodes a protein tyrosine kinase, is involved in protein tyrosine phosphorylation [49,50], a known process that occurs in sperm capacitation [51]. Several proteins function with spe-8-mediating signaling pathways, which promote motility [52,53]. It has been suggested that Zn^2+^ may initiate the spe-8 signaling cascade, leading to sperm activation [54,55].

Zipt-7.1 is a transmembrane protein localized within intracellular organelles [56,57] and, together with *spe*-8, regulates the release of Zn^2+^ from internal stores. The released Zn^2+^ in the cytoplasm activates zinc-regulated proteins that develop motility. Studies of zinc transporters revealed that the deletion of zipt-7.1 causes sterility [58]. ZIP9 serves as a Zn^2+^ transporter associated with the G-protein Gnα11, which mediates testosterone signaling in murine spermatogenic cells [59]. During ejaculation, Zn^2+^ is transported into the nucleus [16], which is important for chromatin stabilization [60,61]. Thus, Zn^2+^ acts as a second messenger that modulates sperm functions, including motility and capacitation. This suggests that intracellular Zn^2+^ levels should be well-controlled by zinc transporters localized at intracellular membranes and in the cell plasma membrane, which import Zn^2+^ from the external environment [62].

### 2.2. Involvement of Zn^2+^ in Sperm Motility

The impact of zinc on sperm motility has been investigated in many vertebrate and invertebrate species. It has been reported that Zn^2+^ in human seminal plasma decreases sperm motility [63] and that Zn^2+^ removal, by binding to a protein named semenogelin, enhances motility [64]. However, in sea urchin, a treatment with ethylenediamine tetra acetic acid (EDTA), a bivalent metal ion chelator, inhibits sperm motility, an effect that was reversed by the addition of Zn^2+^ [65]. In *C. elegans*, the zinc released within cells acts as a messenger in a signaling pathway to promote mobility acquisition [62]. Thus, extracellular Zn^2+^ affects sperm motility, but whether its effect is inhibitory or stimulatory seems to be species- and concentration-dependent, whereby relatively low Zn^2+^ concentrations stimulate motility, whereas high Zn^2+^ inhibits sperm motility.

Furthermore, it has been shown that Zn^2+^ is present in sperm mitochondria and along the flagella [66,67] and is predominantly localized in the outer dense fibers (ODF) [68,69]. The binding of Zn^2+^ to cysteine-sulfhydryl of ODF during spermatogenesis [70] protects it from premature oxidation [71]. Reduction in the intracellular concentration of Zn^2+^ during epididymal transit enables sulfhydryl oxidation and stiffening of the ODF to allow the development of progressive motility [71] and the subsequent hyper-activated motility (HAM) during sperm capacitation [72]. Hyper-activated motility is a form of sperm motility developed during sperm capacitation at the site of fertilization. This movement is characterized by an intensive and asymmetric beating of the middle and principal pieces of the flagella, which engender a strong driving force to infiltrate the extracellular matrix of oocytes [73]. It has been demonstrated that low concentrations of Zn^2+^ (5–10 µM) stimulate hyper-activated motility in human sperm under capacitation conditions, whereas, at 30-µM Zn^2+^, no stimulation was observed [74]. We also know that relatively high concentrations of Zn^2+^ (in the mM range) in the semen are inhibitory to sperm function, whereas, in the female reproductive tract, the concentration of Zn^2+^ is much lower (1.0–1.5 µM) [75]. This low concentration of zinc allows the occurrence of normal sperm capacitation, including hyper-activated motility, leading to a physiological acrosome reaction and successful fertilization.

**Figure 1:**
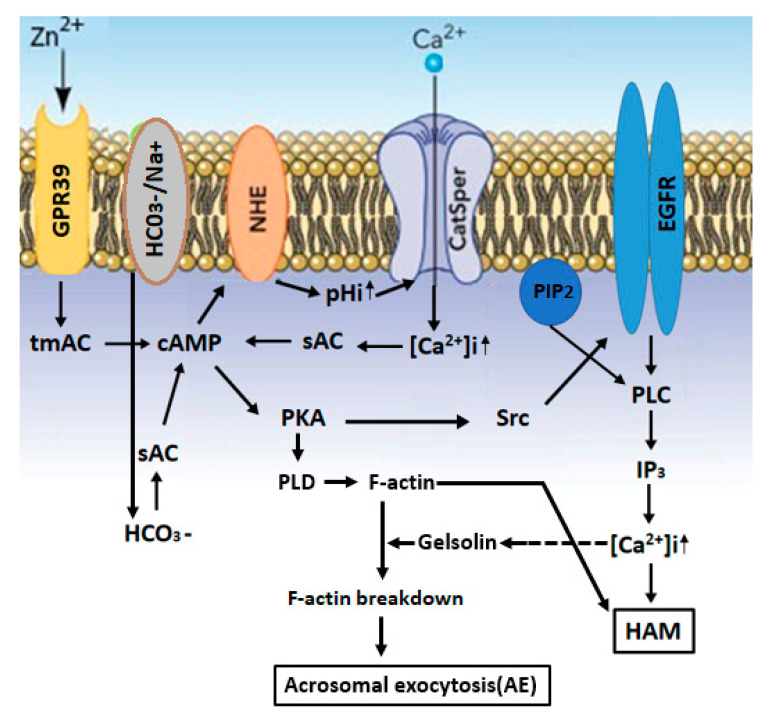
A model describing the mechanisms that mediate the stimulation of hyper-activated motility (HAM) and acrosomal exocytosis (AE) by Zn^2+^: Zn^2+^ binds and activates GPR39, which activates the tmAC to catalyze cAMP production. The NHE (Na^+^/H^+^-exchanger) is activated by cAMP, leading to increased pHi and the activation of CatSper, resulting in an increase in [Ca^2+^]_i_, which, together with HCO_3_^−^, activates sAC. The increase in [cAMP]_i_ causes PKA activation, followed by activation of the Src-epidermal growth factor receptor-Pphospholipase C (Src-EGFR-PLC) cascade, resulting in inositol-triphosphate (IP_3_) production, which mobilizes Ca^2+^ from the acrosome, causing a further increase in [Ca^2+^]_i_ and the development of hyper-activated motility. PKA also activates PLD1 leading to F-actin formation during capacitation. Prior to the AE, PLC induces phosphadidylinositol-4,5-bisphosphate (PIP_2_) hydrolysis, leading to the release of the actin-severing protein gelsolin to the cytosol, which is activated by Ca^2+^, resulting in F-actin breakdown and acrosomal exocytosis (AE).

Motility initiation after sperm ejaculation, and the development of hyper-activated motility during sperm capacitation, are both dependent on intracellular alkalization [76]. Excessive Zn^2+^ concentration (0.1 mM) inhibits sperm motility [77]. High concentrations of Zn^2+^ (0.2 mM), which compromise capacitation and hyper-activated motility, also inhibit the voltage-gated H^+^ channel Hv1, localized in the sperm tail and responsible for sperm cytoplasmic alkalization [78,79] and the regulation of human sperm tail rotation and hyper-activated motility [80]. The cytoplasmic alkalization leads to the activation of the sperm-specific Ca^2+^ channel CatSper [81], which is localized in the flagellum and mediates the development of capacitation-dependent hyper-activated motility [82] (see Figure 1). It has been shown that the CatSper inhibitor negatively impacts the stimulatory effect of Zn^2+^ on human sperm HAM, indicating that CatSper mediates Zn^2+^-stimulated hyper-activated motility [74]. Hyper-activated motility develops during sperm capacitation [83], a process that depends on protein kinase A (PKA) activity [84]. CatSper-null sperm show normal PKA activity [82,85], indicating that PKA activity is not dependent on CatSper. We found that CatSper inhibition by NNC-55-0396(1S,2S-2-(2-[N-((3-benzimidazole-2-yl)propyl)-N-methyamino]ethyl)-6-fluoro-1,2,3,4-tetrahydro-1-isopropyl-2-naphtylcyclopropanecarboxylate-dihydrochloride hydrate) resulted in PKA inhibition, indicating that PKA activity depends on CatSper activity [74]. This possible contradiction could be answered as follows: Ca^2+^ influx via CatSper causes membrane depolarization, leading to the activation of voltage-gated H^+^ channel H_v_1, which is the predominant alkalization system in human sperm [86]. Thus, the inhibition of CatSper by NNC-55-0396 will cause hyperpolarization, leading to the inhibition of H_v_1 and preventing intracellular alkalization. The increase of intracellular pH will shift the equilibrium of the reaction: CO_2_+H_2_O-→HCO_3_^−^+H^+^ to the left, resulting in decreasing the concentration of bicarbonate and a reduction in sAC/PKA activities. CatSper-null sperm was produced in the mouse, in which intracellular alkalization occurs by Na^+^/H^+^ exchanger (NHE) and H_v_1 does not exist. NHE-null sperm is not motile, and motility could be restored by artificial alkalization of the cytosol [87]. Since sAC activity is defected in NHE-null mice, it was suggested that lower intracellular pH in these cells is responsible for the motility defect [87], and it is possible that intracellular alkalization might activate sAC. Thus, the inhibition of Ca^2+^ influx in CatSper-null sperm would not affect intracellular alkalization, allowing the activation of sAC/PKA. We suggested that Zn^2+^ activates the PKA-Src-EGFR cascade, which is CatSper-dependent [74] (see Figure 1). Since CatSper activation is required for hyperactivation and, ultimately, for male fertility [82], it is not unexpected that zinc would be important for preparing the spermatozoa for hyperactivation and successful fertilization.

### 2.3. Zn^2+^ Mediates Sperm Capacitation and Acrosomal Exocytosis

Extracellular Zn^2+^ affects intracellular signaling pathways through its interaction with the Zn^2+^-sensing receptor (ZnR), also named GPR39 [65]. This receptor was found in the sperm tail and the acrosome [74,88,89], suggesting the possible involvement of Zn^2+^ in sperm motility and acrosomal exocytosis. Our studies showed that Zn^2+^ stimulates bovine sperm acrosomal exocytosis [88], as well as human sperm hyper-activated motility [74], both mediated by GPR39 (see Figure 1). The GPR39 receptors belong to the GPCR family, known to activate trans-membrane adenylyl-cyclase (tmAC). The function of tmAC in mammalian sperm is still controversial [90]. As mentioned above, tmAC is regulated by the G-protein, and the stimulatory G-protein (Gs) is, so far, localized in the sperm head [91], suggesting its possible involvement in the acrosome reaction but probably not in sperm motility. A previous study from our laboratory showed the involvement of two GPCRs in bovine sperm, angiotensin II-receptor (AngII-R) and lysophosphatidic acid-receptor, in sperm capacitation [92]. Motility and PKA activation in amphibian sperm are also stimulated by tmAC [93]. Human sperm treated with 5-µM Zn^2+^ show a 40% increase in intracellular cAMP levels, which is a necessary event in the capacitation process [88]. Higher concentrations of zinc (20–30 µM) caused a much smaller increase in cAMP cellular levels [74]. These effects of zinc concentrations of cAMP levels are well-correlated with the stimulation of HAM, with a high increase in HAM with 5-µM zinc and lower effect with 20–30-µM zinc [74]. It has been suggested that Zn^2+^ mediates the activity of the two adenylyl-cyclase(AC) isoforms, the soluble AC (sAC), activated by bicarbonate, as well as the trans-membrane AC (tmAC), activated by GPCR, leading to an increase of intracellular cAMP (see Figure 1). Interestingly, the addition of 8Br-cAMP (a membrane-permeable cAMP analog) to human sperm revealed a significant stimulation of hyper-activated motility at relatively low concentrations (10–15 µM), and this stimulatory effect was reduced at 20 µM and disappeared at 30-µM 8Br-cAMP [74]. The similarity of the dose response effect on the hyper-activated motility between zinc and cAMP clearly suggests that zinc mediates the hyper-activated motility by increasing the intracellular cAMP levels. Surprisingly, the stimulatory effect of extracellularly administered 8Br-cAMP on HAM was inhibited by sAC but not by tmAC inhibitors [74], conditions under which the cellular levels of cAMP should not be affected, since 8Br-cAMP was added to the cells. We can explain this result by suggesting that the cAMP supplied to the cells may be excluded from cellular locations at which sAC provides cAMP for HAM. Interestingly, attempts to bypass the need for sAC activity by providing cAMP did not restore fertilization competence of sAC-null sperm [94]. Several studies suggested that cAMP signaling in sperm is compartmentalized to the head and three regions of the tail: the midpiece, principal piece and endpiece [95,96,97,98]. This compartmentalization might be the basis for different cAMP responses of the tail waveform in the proximal and distal regions of the flagella upon bicarbonate (an activator of sAC) induction [99]. The cAMP microdomains are formed due to the distinct flagellar distribution of at least two phosphodiesterase (PDE) isoforms [100], PDE4 and PDE1, which regulate sperm capacitation and motility [84,101,102].

It has been shown that the in vitro addition of high concentrations of Zn^2+^ to bovine [88] and human [74] sperm could lead to the inhibition of several capacitation processes and reduced fertility rate [103]. Zinc has antioxidant activity and may decrease the levels of reactive oxygen species (ROS) [104,105]. It was shown that ROS production is essential for sperm capacitation [106,107]; however, relatively high levels of ROS can harm sperm functions [108]. Thus, low zinc concentrations might be beneficial in reducing excessive levels of ROS, whereas high zinc might decrease ROS to a level that is inhibitory to sperm capacitation [74,109]. The relatively low concentrations of Zn^2+^ (1.0–1.5µM) [75] in the female reproductive tract allows the occurrence of sperm capacitation and the acrosome reaction, leading to fertilization. It has been proposed [110] that zona pellucida (ZP) proteinases implicated in endowing the acrosome reacted spermatozoon with the ability to penetrate the ZP are negatively regulated by Zn^2+^. Sperm can induce Zn^2+^ release from the oocyte cortex [111,112], leading to proteinase inhibition; as a result, sperm that are still bound to the ZP became decapacitated, and polyspermy was prevented. It was also suggested that Zn^2+^ inhibits sperm chemoattraction to the egg induced by oocyte-secreted progesterone in human, mouse and rabbit sperm [113], and the addition of Zn^2+^ (~0.1 mM) to bovine in vitro fertilization (IVF) medium reduces the fertilization rate [114]. Additionally, blockers of Zn^2+^-dependent metalloproteases inhibit sperm passage via the cumulus oophorus in porcine IVF [115].

Matrix metalloproteinases (MMPs) are part of the Zn^2+^-dependent endopeptidase family. The isoform MMP2 is localized to the acrosomal region and tail, and MMP9 is expressed in the tail of human sperm [116,117]. MMP2, together with the proteinase acrosin, are localized to the inner acrosomal membrane, suggesting the possibility of their cooperation in oocyte penetration [118]. We have shown that MMP is involved in the transactivation of the epidermal growth factor receptor (EGFR) by activating GPCRs during sperm capacitation [92].

Low concentrations of Zn^2+^ (in the µM range) increase the in vitro capacitation efficiency [74,88] by activating several proteins during this process, including the PKA, Src, EGFR and phosphatidylinositol-3-kinase (PI3K) [119,120,121,122], leading to intracellular Ca^2+^ mobilization and acrosomal exocytosis (see Figure 1). We suggested the following mechanism that regulates human sperm hyper-activated motility: Zn^2+^ stimulates HAM via CatSper-dependent activation of the adenylyl-cyclase (AC)/cAMP/PKA/Src/EGFR and phospholipase C (PLC) cascade [74] (see Figure 1). In bovine sperm, Zn^2+^ activates the EGFR during capacitation, which is mediated by the activation of tmAC, PKA and Src [88]. The addition of Zn^2+^ to capacitated bovine sperm further stimulates the EGFR and the downstream effectors PI3K, PLC and protein kinase C (PKC), leading to acrosomal exocytosis [88] (see Figure 1). Under physiological conditions, inositol-3-phosphate receptor (IP_3_R) localized in the outer acrosomal membrane and in the redundant nuclear envelope (RNE) are activated by IP_3_ generated from the hydrolysis of phosphadidyl-inositol-4,5-bisphosphate (PIP_2_) by PLC, inducing the release of Ca^2+^ from the acrosome and RNE and promoting the development of HAM [123]. Thus, this cascade can be initiated by Zn^2+^-activated GPR39, leading to PLC activation, as described above.

It is known that the occurrence of spontaneous acrosomal reaction (sAR) before the sperm reaches the proximity of the egg reduces the fertilization rate [124]. Sperm contain several mechanisms that protect it from undergoing sAR [125,126,127,128]. We showed that actin polymerization during sperm capacitation is crucial for preventing sAR [125] and found that the addition of 5-10µM Zn^2+^ to bovine sperm increases actin polymerization and decreases the sAR rate (unpublished). The integrity of the sperm acrosome is likely to be defective in sperm with a high rate of sAR. Accordingly, a dietary supplementation of Zn^2+^ to goats increases sperm acrosome integrity [104], supporting the role of Zn^2+^ in preventing sAR.

### 2.4. Zinc in Assisted Reproductive Techniques 

The effectiveness of assisted reproductive methods has been improved over the last decade. The cryopreservation of sperm under liquid nitrogen is now used in assisted reproduction centers to conserve sperm cells for extended periods of time. However, freezing and thawing processes damage the fertilizing capacity of the sperm due to osmotic effects and oxidative stress. Under cryopreservation, sperm is deprived of the seminal plasma, which contains antioxidants and vitamins that act against free radicals and, thereby, protect the sperm cells [129,130].

Cryopreservation affects sperm cells in many ways: by diminishing the fertilization capacity, reducing motility, altering morphology (such as coiled tails), reducing viability [131], damaging the cell membrane [132] and causing DNA fragmentation [133,134] and loss of mitochondrial function [135].

The addition of antioxidants, such as zinc, in the freezing medium to improve the fertility capacity and preserve the sperm from oxidative damage is becoming increasingly widespread [136,137]. Studies revealed that, after incubation with hydrogen peroxide, the DNA fragmentation percentage in spermatozoa was increased, and the effect was reversed by zinc supplementation to the medium [138].

Recent studies demonstrated the beneficial effects of zinc addition to human ejaculate before cryopreservation on sperm viability and motility after thawing [138,139]. The freezing of human sperm in the presence of 50-µM zinc revealed a 26–184% increase in the number of motile sperm after thawing and a 130% increase in the percentage of progressive motility compared to a control without zinc [139]. Moreover when cells were frozen, thawed and refrozen in the presence of zinc, a considerable increase in motility was observed [139] relative to the first thaw. Moreover, zinc has been reported to preserve genomic integrity [140], chromosomal stability [141,142] and to protect the sperm membrane [143,144] and cell morphology during cryopreservation.

Zinc oxide nanoparticles (ZnONPs), initially developed for drug delivery in cancer research [145], were applied to study sperm preservation during cryopreservation. The ZnONPs seemed to prevent DNA damage and stabilize sperm chromatin [146]. These protective effects were reported to be linked to the creation of a protective layer of ZnONPs around the sperm cell, preventing lipid peroxidation at the membrane [146].

Zinc-deficient nutrition causes a low quality of sperm and male infertility [11], and the presence of zinc in the diets of humans [27] and domestic animals [28,29,30,147] is required for the achievement of an optimal fertility rate. Nevertheless, recent clinical studies investigated the effect of dietary supplements containing zinc and showed that zinc supplementation does not appear to improve pregnancy rates, sperm counts or sperm function [148]. The addition of zinc in the micromolar range to an in vitro fertilization medium can contribute to improve sperm motility and capacitation, which will increase the fertilization rate.

## 3. Conclusions

An adequate Zn^2+^ concentration in the seminal plasma is needed for normal sperm function and fertilization; however, highly the toxic content of Zn^2+^ may have a negative effect on sperm quality. Although many studies prove the association between seminal plasma Zn^2+^ concentration and sperm physiology, it certainly cannot be said that seminal Zn^2+^ deficiency causes infertility. The addition of zinc in the micromolar range to a sperm medium in vitro can contribute to the amelioration of sperm motility and capacitation to fertilize the oocyte. Zinc supplementation should be considered as a good player for the improvement of in vitro-assisted reproduction procedures, although Zn^2+^ dietary supplementation has not already been proved to ameliorate pregnancy rate in humans. Altogether, the currently available data revealed the importance of zinc ions for male fertility, which could be important to improve human reproductive health and the reproductive efficiency in agriculturally important livestock species. Further studies in fertile and infertile men are required to prove this claim, and the determination of the seminal Zn^2+^ level in these men will help to solve this question.

## Abbreviations

MDPI	Multidisciplinary Digital Publishing Institute
DOAJ	Directory of open access journals
TLA	Three-letter acronym
LD	linear dichroism
